# What can be lost? Genomic perspective on the lipid metabolism of *Mucoromycota*

**DOI:** 10.1186/s43008-023-00127-4

**Published:** 2023-11-06

**Authors:** Blanka Sokołowska, Małgorzata Orłowska, Alicja Okrasińska, Sebastian Piłsyk, Julia Pawłowska, Anna Muszewska

**Affiliations:** 1 Institute of Biochemistry and Biophysics, Polish Academy of Sciences, Pawinskiego 5A, 02-106 Warsaw, Poland; 2 Faculty of Biology, Biological and Chemical Research Centre, Institute of Evolutionary Biology, University of Warsaw, Zwirki i Wigury 101, 02-089 Warsaw, Poland

**Keywords:** Protein family, Lipid metabolism, Ergosterol, Fatty acid, Early diverging fungi, Plant-associated fungi

## Abstract

*Mucoromycota* is a phylum of early diverging fungal (EDF) lineages, of mostly plant-associated terrestrial fungi. Some strains have been selected as promising biotechnological organisms due to their ability to produce polyunsaturated fatty acids and efficient conversion of nutrients into lipids. Others get their lipids from the host plant and are unable to produce even the essential ones on their own. Following the advancement in EDF genome sequencing, we carried out a systematic survey of lipid metabolism protein families across different EDF lineages. This enabled us to explore the genomic basis of the previously documented ability to produce several types of lipids within the fungal tree of life. The core lipid metabolism genes showed no significant diversity in distribution, however specialized lipid metabolic pathways differed in this regard among different fungal lineages. In total 165 out of 202 genes involved in lipid metabolism were present in all tested fungal lineages, while remaining 37 genes were found to be absent in some of fungal lineages. Duplications were observed for 69 genes. For the first time we demonstrate that ergosterol is not being produced by several independent groups of plant-associated fungi due to the losses of different ERG genes. Instead, they possess an ancestral pathway leading to the synthesis of cholesterol, which is absent in other fungal lineages. The lack of diacylglycerol kinase in both *Mortierellomycotina* and *Blastocladiomycota* opens the question on sterol equilibrium regulation in these organisms. Early diverging fungi retained most of beta oxidation components common with animals including Nudt7, Nudt12 and Nudt19 pointing at peroxisome divergence in Dikarya. Finally, *Glomeromycotina* and *Mortierellomycotina* representatives have a similar set of desaturases and elongases related to the synthesis of complex, polyunsaturated fatty acids pointing at an ancient expansion of fatty acid metabolism currently being explored by biotechnological studies.

**Supplementary Information:**

The online version contains supplementary material available at 10.1186/s43008-023-00127-4.

## INTRODUCTION

*Mucoromycota* is a phylum of early diverging fungi (EDF) lineages, consisting mostly of plant-associated terrestrial fungi characterized by aseptate mycelium and the formation of zygospores during sexual reproduction (James et al. 2020). It is divided into three subphyla: *Mucoromycotina*, *Mortierellomycotina*, and *Glomeromycotina* (James et al. 2020). Representatives of *Mucoromycotina* are known for the ability to produce polyunsaturated fatty acids (PUFAs) with chain lengths up to 18 carbon atoms, with the best studied one being gamma-linoleic acid (GLA) (Kosa et al. 2018). *Umbelopsis isabellina* being one of the most important industrially used producers of this compound (Klempova et al. 2013). On the other hand, *Mortierellomycotina* groups some of the most oleaginous fungi that are able to synthesize PUFAs with chain lengths up to 20 carbon atoms (Chang et al. 2022; Klempova et al. 2013). Due to the potential industrial applications (e.g. biodiesel production), there is still increasing interest in researching their lipid metabolism (Athenaki et al. 2018).

Lipids are a diverse group of macromolecules, which can all be characterized by their insolubility in polar solvents and solubility in non-polar solvents (Akoh and Min 2002). They constitute up to 40% of cell mass in eukaryotes (Muro et al. 2014) and around 5% of the genes are involved in their biosynthesis (Sud et al. 2007; van Meer et al. 2008). Lipids are used as a storage material, source of energy, signaling molecules, and are one of crucial components of membranes (Subramaniam et al. 2011). Lipid metabolism is a part of core cell metabolism and is essential for cell division, growth, and reproduction.

**Fig. 1:**
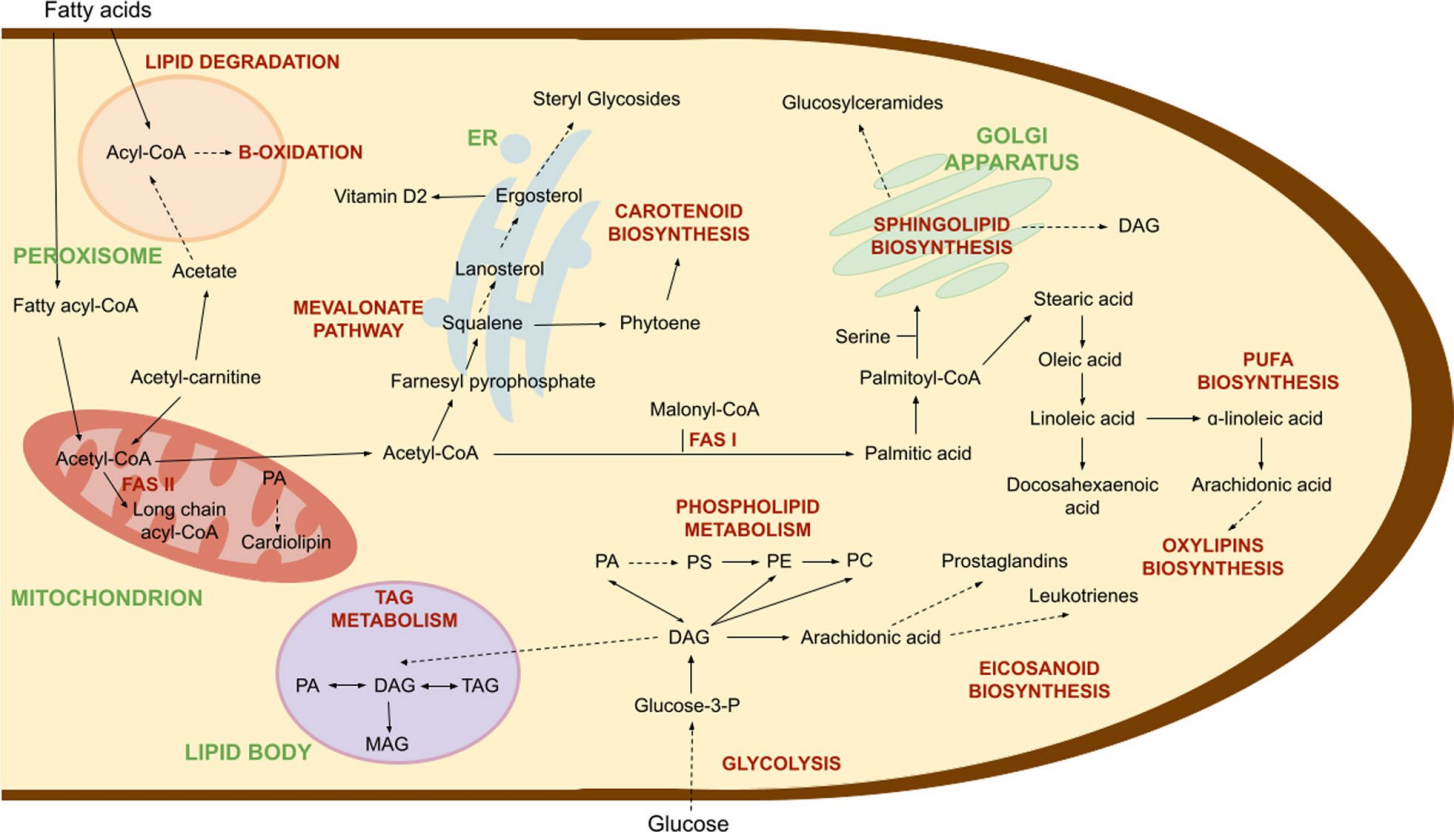
Cellular localization of lipid metabolic pathways in Fungi

Although in cell biology, lipids are commonly associated with the plasma and organelle membranes, lipid metabolic pathways of Fungi, involve core fatty acid metabolism, fatty acid elongation and desaturation processes, oxylipins and sterol biosynthesis, sphingolipid and phospholipid metabolism, triacylglycerol and lipid bodies formation, peroxisome and lipid degradation, carotenoid metabolism, which take place in different cellular compartments (Fig. 1). Fatty acid biosynthesis, cardiolipin production and beta-oxidation occurs in mitochondria where lipids serve as energy source and can modulate the responses of other organelles (Mayr 2015). Most enzymes involved in lipid synthesis are located in the endoplasmic reticulum (ER). Extracellular signals modulate the activity of these enzymes and allow the ER to respond to the changing environment. Membrane lipid biosynthesis (e.g. ergosterol, steryl glucosides) by the ER is essential for growth, proliferation, and also maintaining the homeostasis of the cell (Jacquemyn et al. 2017). Carotenoids, which protect the cell against oxidative stress and UV radiation, are another important product of ER metabolism (Avalos and Carmen Limón 2015; Klempova et al. 2013). The Golgi complex acts as a central station for sorting and transporting lipids. It is also involved in the biosynthesis of sphingolipids that constitute 10–20% of all cellular lipids (Goto et al. 2020). Peroxisomes, often relegated as the “cellular vacuum cleaners”, are also responsible for beta-oxidation, lipid biosynthesis, and lipid degradation (Lodhi and Semenkovich 2014). Lipid droplets are storage organelles with a unique architecture consisting of a hydrophobic (fatty) core mostly made of neutral storage lipids, including triacylglycerols (TAGs), sterol esters, and their intermediates (Koch et al. 2014). Although they originate from the ER, throughout their lifecycle, lipid droplets come in contact with other organelles, such as the mitochondria, peroxisomes, and vacuoles (Schuldiner and Bohnert 2017). Alternating between lipolysis and lipophagy (the selective autophagic degradation of lipid droplets), the lipid droplets’ metabolic activity depends on the availability of nutrients and changes in the cellular metabolism (Olzmann and Carvalho 2019).

Fatty acid biosynthesis also occurs in the cytoplasm. PUFAs are considered to be crucial sources of metabolic energy, significant structural components of membrane phospholipids, and precursors of the eicosanoid signaling molecules, such as prostaglandins, thromboxanes, and leukotrienes (Dourou et al. 2017; Noverr et al. 2002).

Here, we present a comprehensive overview of genes and pathways of the lipid metabolism in EDF, focusing on major constituents of membranes. We trace the evolution of the *Mucoromycota* lipidome components, and show the ubiquity of the core of lipid metabolism and peculiarities with limited taxonomic distribution likely contributing to the ecology of particular lineages.

## RESULTS

Genes encoding proteins involved in lipid metabolism are highly conserved in evolution, due to their significance in physiology and homeostasis of the cell. In this study 202 genes were gathered and analyzed among 37 strains of *Mucoromycota*, three species of *Ascomycota* and two representatives of *Blastocladiomycota*, *which* were used as a reference outgroup.

### Core fatty acid metabolism

Fatty acid synthase (FAS) is responsible for de novo biosynthesis of fatty acids from acetyl-CoA and malonyl-CoA (KEGG pathway: map00061). There are 2 types of FAS enzymes in fungal cells: cytoplasmic FAS-I type and mitochondrial FAS-II type. Cytoplasmic FAS-I is a multienzyme made of alpha and beta subunits harboring many different enzymatic properties, coded by FAS2 and FAS1 genes, respectively (Fischer et al. 2020). *Glomeromycotina* representatives lack both FAS1 (*S. cerevisiae* accession: P07149) and FAS2 (*S. cerevisiae* accession: P19097) genes. This is consistent with the documented loss of cytoplasmic FAS-I type in the *Glomeromycotina* subphylum (Tang et al. 2016).

We also identified several gene duplications in the *Mucorales* group within the *Mucoromycotina* subphylum. ACP1 (*S. cerevisiae* accession: P32463) gene duplication is prevalent in the *Mucorales* order except for the *Cunninghamellaceae*, with additional duplication in *Mucoraceae*. MCT1 (*S. cerevisiae* accession: Q12283) gene duplication was found in the *Cunningamellaceae* family.

### Fatty acid elongation and desaturation

Taking place in cytoplasm, elongation of fatty acid proteins 1, 2, and 3 are components of membrane bound medium-chain (ELO1 coded protein) and long-chain (ELO2 and ELO3 coded proteins) fatty acid elongation system (KEGG pathway: map00062). Elongation of fatty acid proteins 1 is a component of elongase 1, which extends 12–16-carbon fatty acyl-CoAs, such as lauroyl-CoA, to 14–18-carbon fatty acids by incorporation of malonyl-CoA (Schneiter et al. 2000). Elongation of fatty acids protein 2 is a component of elongase 2 and produces up to 22-carbon very long-chain fatty acids (Oh et al. 1997). Elongation of fatty acids protein 3 is a component of elongase III and synthesizes the 20–26-carbon very long-chain fatty acids (VLCFA) from long-chain fatty acid precursors and is involved in ceramide and inositol sphingolipid biosynthesis (Rössler et al. 2003). Genes coding ELO1, ELO2, and ELO3 proteins have clustered together on the phylogenetic tree, meaning that their sequences are closely related to each other. *Umbelopsidales* representatives possess four copies of ELO-like gene and *Mucorales* have two copies with additional expansions and duplication in the *Mucorinae* group. *Glomeromycotina* representatives have two copies and *Mortierellomycotina* representatives vary from two to four copies in their genomes. Very long-chain fatty acid transport protein FAT1 (*S. cerevisiae* accession: P38225) has two separate functions; it is necessary for import of long chain fatty acids (LCFAs) and has acyl-CoA synthetase activity responsible for activating the very long chain fatty acids (VLCFAs) C20-C26 by esterification of the fatty acids into CoA-thioesters. Further those esterified products can be used for incorporation of LCFAs into phospholipids or their degradation (Choi and Martin 1999; van Roermund et al. 2012). Gene duplication has occurred in *Mucorales* (with several gene expansions) and *Umbelopsidales* groups within the *Mucoromycotina* subphylum, as well as in the *Mortierellomycotina* subphylum. *Blastocladiomycota* representatives seem to lack FAT1 gene.

Fatty acid desaturation is a process of producing a variety of unsaturated and polyunsaturated fatty acids containing a double carbon bond (KEGG pathway: map01040). Acyl CoA desaturase 1 Ole1 (*S. cerevisiae* accession: CAA96757) is a stearoyl-CoA desaturase that introduces the first cis double bond at delta-9 position into saturated fatty acyl-CoA substrates, such as palmitoyl-CoA and stearoyl-CoA (Stukey et al. 1990). *Glomeromycotina* and *Mortierellomycotina* representatives possess ancestral gene duplication with gene expansion in *Lobosporangium* sp. Additional gene duplication happened in the *Mucorales* (with several gene expansions in the *Rhizopus, Lichtheimia,* and *Phycomyces* genera). One of the duplicated Ole1-like proteins in *Glomeromycotina* may produce palmitvaccenic acid (Δ11-cis-hexadecenoic acid), instead of oleic acid (Brands et al. 2020).

**Fig. 2:**
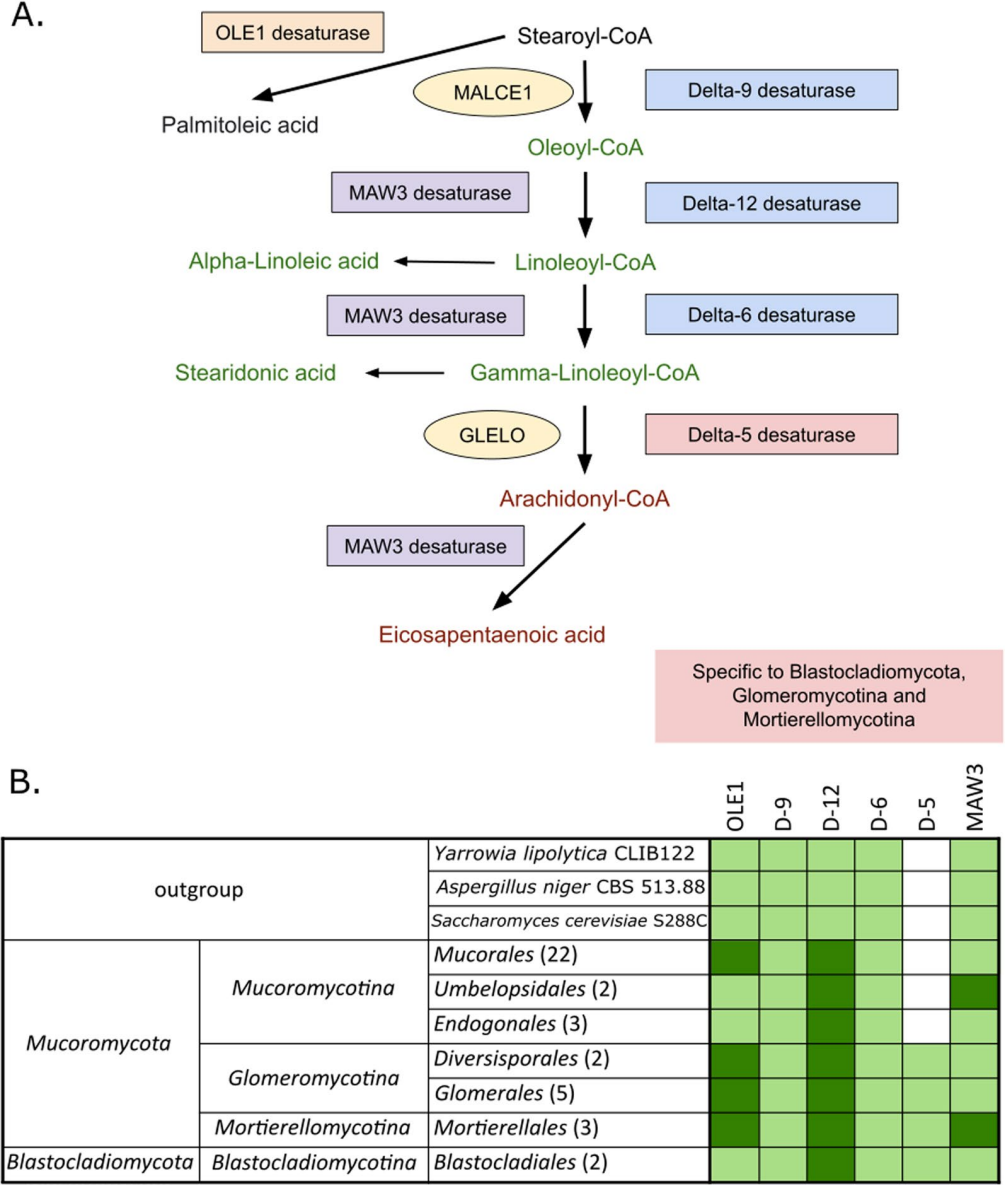
Schematic metabolic map of fatty acid desaturation in EDF (**A**)**.** and a table showing the distribution of genes coding fatty acid desaturases involved in PUFA production among tested fungal lineages (**B**). Lighter green indicates presence of tested genes, while darker green indicates additional duplications

Polyunsaturated fatty acids (PUFAs) serve as metabolic energy, precursor for membrane phospholipids, oxylipins, and eicosanoids, such as leukotrienes and prostaglandins (Kikukawa et al. 2018). One of the most industrially important PUFAs in fungi is arachidonic acid (ARA, C20:4n-6), synthesized by desaturases delta-9 (D9), delta-12 (D12), delta-6 (D6) and delta-5 (D5) (Kikukawa et al. 2018). D6 desaturase is also used for gamma-linoleic acid production (GLA, 18:3n-6) (Fig. 2). Desaturase D5 (*L. elongata* accession: OAQ35213) is specific for *Blastocladiomycota, Mortierellomycotina,* and *Glomeromycotina*, while it is not present in the *Mucoromycotina* and in the Dikarya. *Mucoromycota* representatives show duplication in the gene coding D9 desaturase (*M. alpina* accession: CAL69820), except for *Umbelopsidales* and *Endogonales*, with multiple gene expansions across the whole phylum. Duplications were noted in the gene coding D12 desaturase (*L. elongata* accession: OAQ31753) in the whole *Mucoromycota* with several gene expansions across the whole group. Another protein involved in the PUFA production is ER-localized acyl-lipid omega-3 desaturase MAW3 (*M. alpina* accession: Q59J82), which uses both 18-carbon and 20-carbon n-6 polyunsaturated fatty acids. Gene duplication has occurred in the *Mortierellomycotina* and in the *Umbelopsidales* order within the *Mucoromycotina*. Gene expansion is also prominent in the *Phycomycetaceae* family within the *Mucorales*.

**Fig. 3:**
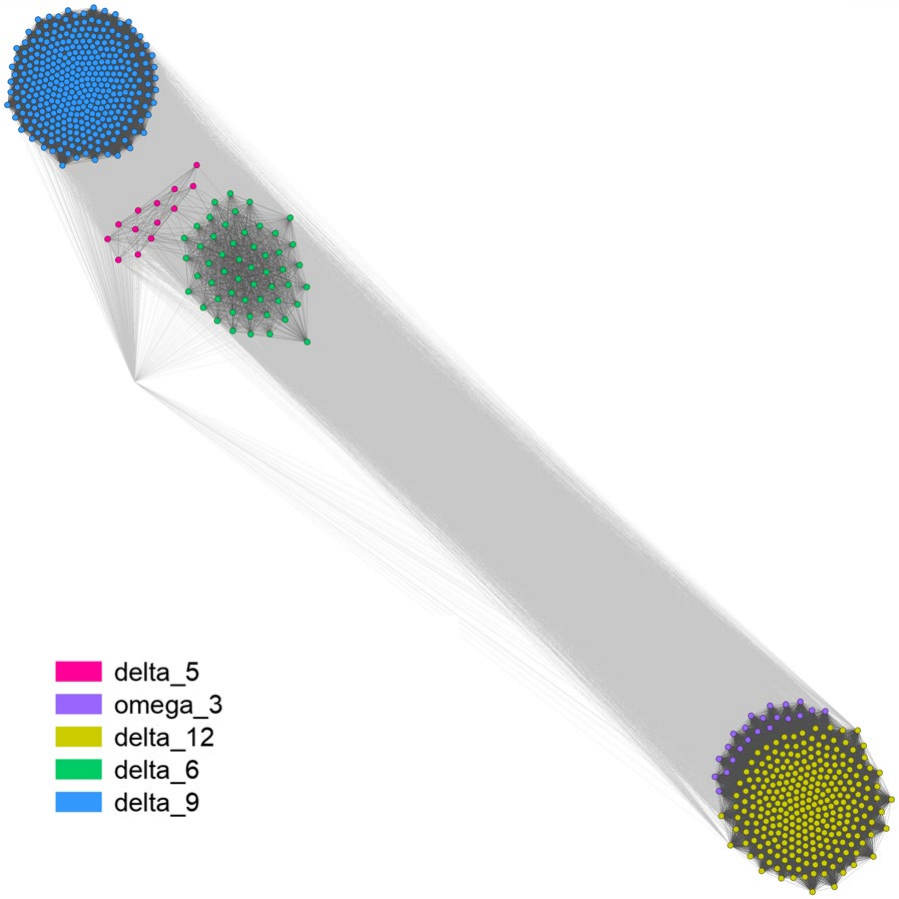
Desaturase sequence clustering shows similarities based on blast scores (CLANS)

Fatty acid desaturases are characterized by a FA_desaturase domain (PF00487) often followed by a cytochrome domain (PF00173). Subfamilies of desaturases are labeled based on the position of double bond insertion. On the sequence level, identified EDF desaturases are grouped according to the position on which they operate (Fig. 3). There is clear sequence similarity between D5 and D6 desaturases, whereas D9 and D12 are clearly separated from all of the remaining ones. There is a paralog of D12 desaturases with omega-3 activity discernible from the canonical D12 desaturases. These omega-3 desaturases are restricted to *Glomeromycotina*, while omega-9 desaturases occur in *Gigasporales*. *Mortierellomycotina* omega-3 desaturases (MAW3) are clustered together with D12 sequences. Acyl-CoA desaturases (Ole1) are grouped together with other D9.

Oxylipin producing enzymes labeled as ppoA, ppoB and ppoC share an An_peroxidase (PF03098) protein domain followed by a p450 cytochrome domain (PF00067). This architecture is present both in oxylipins and animal type heme peroxidases (e.g. *Rhizophagus irregularis* A0A2H5TT10). An_peroxidase domain seems to be limited to *Ascomycota*, while p540 cytochrome domain is conserved among EDF but not related to lipid metabolism. Oleate delta-12 desaturase OdeA (*Aspergillus nidulans* accession: Q9HF05) and Acyl-CoA desaturase SdeA (*A. nidulans* accession: Q8NJU5) responsible for the central steps of oxylipin production are distributed among *Sordariomycetes* and *Eurotiomycetes* representatives. They share a fatty acid desaturase domain FA_desaturase (PF00487) with other fatty acid desaturases and seem to be *Ascomycota* specific. Those oxylipin desaturates are a subtype of fatty acid desaturases and that’s why they are grouped together with D12 desaturates involved in PUFA production (Fig. 3). These observations are particularly puzzling in the light of reports on 3-hydroxy oxylipin presence in *Pilobolus* sp. already in 2001 (Kock et al. 2001), but not reported after 2003 (Kock et al. 2003). Possible explanations include the recruitment of other desaturases to oxylipin formation in non-Dikarya representatives.

### Sphingolipid metabolism

Sphingolipids are another crucial element of eukaryotic cell membranes (KEGG pathway: map00600). The basic structure of fungal sphingolipids consists of a LCB backbone amide linked to a fatty acid at C2 position and ester linked at a C1 position to a polar head group (Del Poeta et al. 2015). In addition to their basic structural function, they are involved in hyphae formation, growth, determining cell polarity, and virulence (Mota Fernandes and Del Poeta 2020). Lipid rafts formed by sphingolipids and sterols have been identified in fungal plasma membranes (Alvarez et al. 2007).

**Fig. 4:**
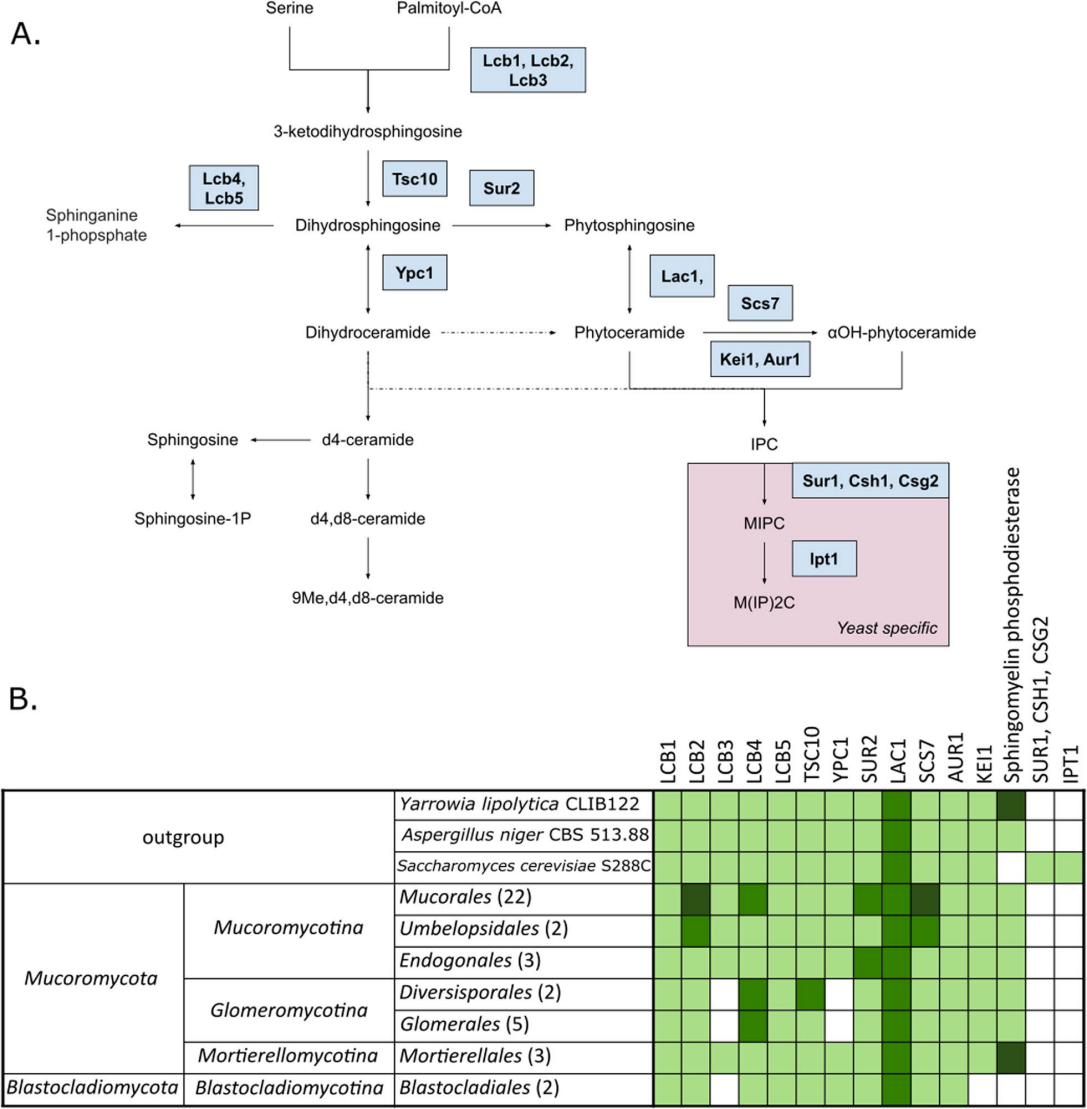
Metabolic map of sphingolipid synthesis based on yeast model (**A**) and a table showing the distribution of genes encoding proteins involved in the sphingolipid metabolism (**B**). Lighter green indicates presence of tested genes, while darker green indicates additional duplications

Serine palmitoyltransferase subunits (Lcb1 *S. cerevisiae* accession: P25045 and Lcb2 *S. cerevisiae* accession: P40970) form serine palmitoyltransferase (SPT) that catalyzes the committed step of sphingolipid biosynthesis (Fig. 4), the condensation of serine with palmitoyl-CoA consequently forming a long chain base 3-ketosphinganine (Nagiec et al. 1994). Dihydrosphingosine 1-phosphate phosphatase LCB3 (*S. cerevisiae* accession: P47013) is required for efficient ceramide synthesis from exogenous sphingoid bases (Qie et al. 1997). Sphingoid long chain base kinase 4 (Lcb4 *S. cerevisiae* accession: Q12246 and its paralog Lcb5 *S. cerevisiae* accession: Q06147) is responsible for the phosphorylation of the long chain sphingoid bases dihydrosphingosine (DHS or sphinganine) and phytosphingosine (PHS) (Nagiec et al. 1998). Gene duplication has occurred in the LCB2 gene in the *Mucoromycotina* (except for *Endogonales* order) with additional copy in the *Mucorales*. *Blastocladiomycota* and *Glomeromycotina* have lost the LCB3 gene. Gene duplication has been shown in the LCB4 gene in the *Glomeromycotina* and in the *Mucorales* with several gene expansions.

Sphingomyelin phosphodiesterase (*M. circinelloides* accession: S2JQ58, homolog to human SMPD1 protein, *Homo sapiens* accession: P17405) converts sphingomyelin to ceramide in humans (Schuchman et al. 1991). The gene encoding this protein was detected in the genomes of most of the fungal species studied, with the exception of *Blastocladiomycota* representatives and *S. cerevisiae*. Duplication of this gene was noted in the *Mortierellomycotina* subphylum, with gene expansion (7 copies) present in *Linnemannia elongata*.

Ceramide synthase LAC1 (*S. cerevisiae* accession: P28496, redundant with its paralog LAG1) is a component of the ceramide synthase complex, responsible for C26-CoA-dependent ceramide synthesis using a fatty acid and sphinganine as substrates (Guillas et al. 2001). Dikarya, *Mucoromycota*, and *Blastocladiomycota* all have two copies of gene coding Lac1 protein. Alkaline ceramidase Ypc1 (*S. cerevisiae accession*: P38298 and its paralog Ydc1 *S. cerevisiae* accession: Q02896), which was lost in *Glomeromycotina*, hydrolyzes phytoceramide and dihydroceramide into phytosphingosine or dihydrosphingosine (Voynova et al. 2014). Gene expansion in *Diversisporales* had been noted in the TSC10 gene coding 3-ketodihydrosphingosine reductase (*S. cerevisiae* accession: P38342), which catalyzes the reduction of 3-ketodihydrosphingosine (KDS) to DHS (Beeler et al. 1998). Sphingolipid C4-hydroxylase Sur2 (*S. cerevisiae* accession: P38992) is responsible for the conversion of sphinganine to phytosphingosine (Grilley et al. 1998). Gene duplication has occurred in the *Mucoromycotina* (except for *Umbelopsidales* order). Ceramide very long chain fatty acid hydroxylase Scs7 (*S. cerevisiae* accession: Q03529) is involved in the hydroxylation of sphingolipid-associated VLCFAs, dihydroceramides and phytoceramides presumably at C-2 position (Haak et al. 1997). Within the *Mucoromycotina* subphylum *Umbelopsidales* were found to possess 2 copies and *Mucorales* representatives possess 3 copies of SCS7 gene.

The inositol phosphorylceramide (IPC) synthase catalyzes the addition of an inositol phosphate group to ceramide, which is a crucial step in sphingolipid biosynthesis. It is composed of regulatory subunit Kei1 (*S. cerevisiae* accession: Q06346) and catalytic subunit Aur1 (*S. cerevisiae* accession: P36107) (Sato et al. 2009; Tani & Kuge 2010). AUR1 is duplicated in *Mucor* spp. and KEI1 gene is duplicated in *Rhizophagus irregularis*, *Mucor* spp., and *Rhizopus microsporus* ATCC 52813. KEI1 subunit seems to be absent in aquatic fungal lineages, such as *Blastocladiomycota* and *Chytridiomycota*, despite the essential function of the holoenzyme ICP in sphingolipid biosynthesis.

*Saccharomycotina* specific genes like Inositolphosphotransferase 1 Ipt1 (*S. cerevisiae* accession: P38954), Mannosyl phosphorylinositol ceramide synthase Sur1 (*S. cerevisiae* accession: P33300), Csh1 (S. cerevisiae accession: P38287) and Csg2 (*S. cerevisiae* accession: P35206) are not present in studied organisms.

### Phospholipid metabolism

**Fig. 5:**
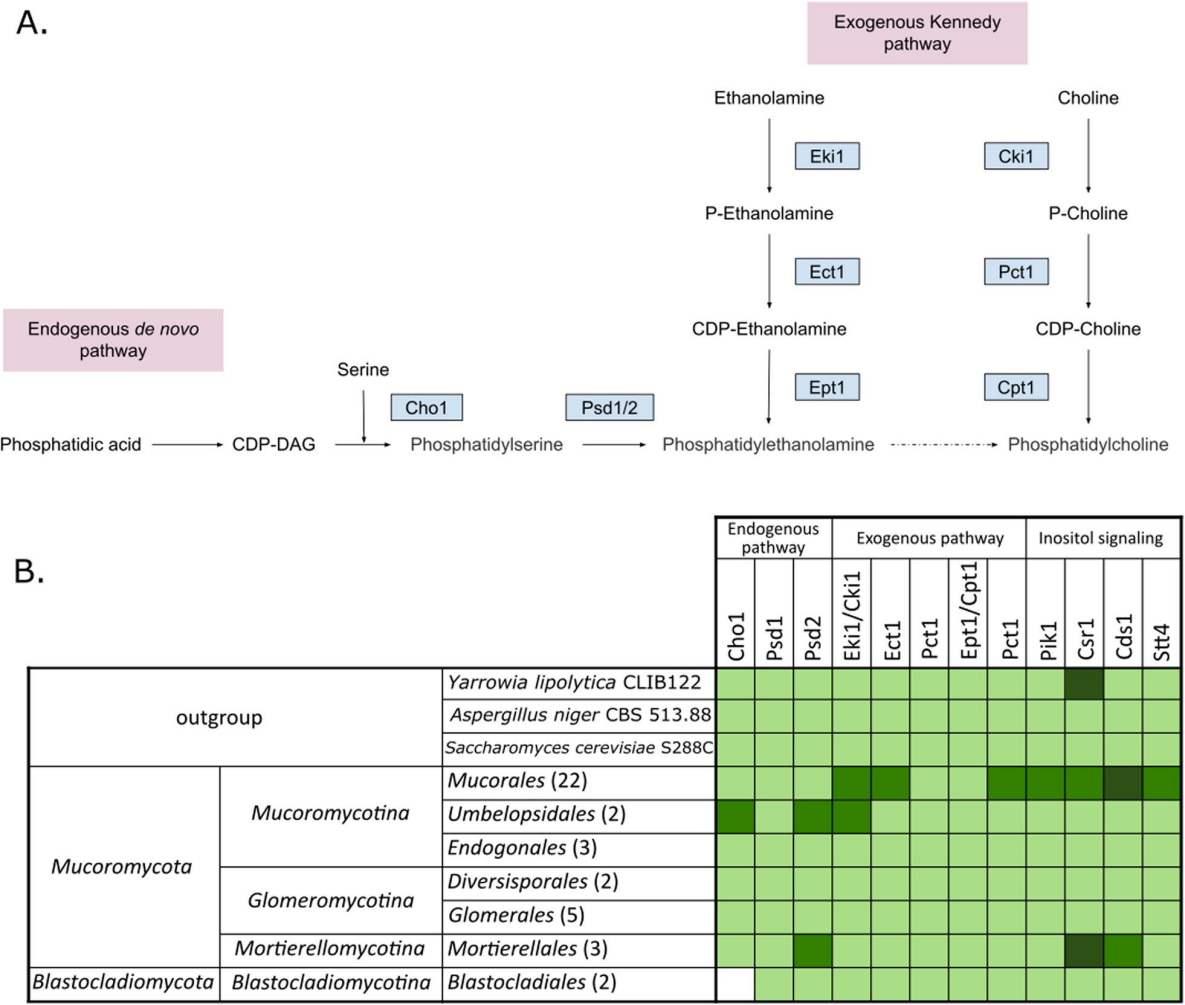
Metabolic map of phospholipid metabolism in yeast model. The dotted arrow shows several intermediate steps that are not shown in the figure (**A**) and a table showing the distribution of genes encoding proteins involved in the phospholipid metabolism (**B**). Lighter green indicates presence of tested genes, while darker green indicates additional duplications

Phospholipids are essential compounds of all cell membranes, crucial for membrane curvature formation (Wang et al. 2021) (KEGG pathway: map00564). Phosphatidic acid (PA) plays crucial role in the phospholipid biosynthesis and serves as the precursor for all phospholipids synthesized via CDP-diacylglycerol (CDP-DAG), CDP-choline and CDP-ethanolamine pathways (Han & Carman 2004) (Fig. 5).

#### Endogenous de novo pathway of phospholipid synthesis

Phosphatidylserine (PS) decarboxylase proenzyme 2 Psd2 (*S. cerevisiae* accession: P53037) is involved in phosphatidylethanolamine biosynthesis from CDP-diacylglycerol (Birner et al. 2001). Gene duplication has occurred in *Umbelopsidales*, as well as *Mucor* spp. and *Mortierellomycotina* subphylum. CDP-diacylglycerol–serine O-phosphatidyltransferase Cho1 (*S. cerevisiae* accession: P08456) catalyzes the first step of phosphatidylethanolamine (PE) biosynthesis from CDP-DAG (Bae-Lee and Carman 1984). *Blastocladiomycota* have lost the CHO1 gene. Gene duplication has occurred in the *Umbelopsidales*.

#### Exogenous Kennedy pathway

Ethanolamine kinase EKI1 (*S. cerevisiae* accession: Q03764) is responsible for the ethanolamine phosphorylation to phosphoethanolamine, which is a part of CDP-ethanolamine pathway (Kim et al. 1999). It has grouped together with its paralog, choline kinase CKI1 (*S. cerevisiae* accession: P20485), which catalyzes the phosphatidylcholine biosynthesis by the CDP-choline pathway (Kim et al. 1998). Gene duplication has occurred in *Umbelopsidales* and all *Mucorales* families except *Cunninghamellaceae*. Choline-phosphate cytidylyltransferase PCT1 (*S. cerevisiae* accession: P13259) is responsible for the first step of CDP-choline pathway, which is a part of phosphatidylcholine biosynthesis from phosphocholine (Dowd et al. 2001). Gene duplication is present in the *Mucorales* order, except for the *Lichtheimiaceae* family.

#### Inositol signaling

There are several enzymes involved in inositol signaling, but only a few showed differences in distribution among fungal species. Phosphatidylinositol 4-kinase Stt4 (*S. cerevisiae* accession: P37297) acts on phosphatidylinositol (PI) in the first committed step in the production of inositol 1,4,5,-trisphosphate, which is a secondary messenger (Audhya et al. 2000). Gene duplication of STT4 is prevalent in the *Mucorales* group. Phosphatidylinositol 4-kinase Pik1 (*S. cerevisiae* accession: P39104) is also involved in the same process (Audhya et al. 2000; Flanagan et al. 1993). Gene duplication is present in the *Mucorinae*. Phosphatidylinositol transfer protein Csr1 (*S. cerevisiae* accession: Q06705) stimulates phosphoinositide synthesis via the Stt4 phosphatidylinositol 4-kinase. It also inhibits Fas activity in response to heme and oleic acid starvation, preventing the accumulation of saturated fatty acids (Desfougères et al. 2008). Gene duplication is prevalent in *Mucorales* representatives. *Mortierellomycotina* has multiple copies of the CSR1 gene. Phosphatidate cytidyltransferase Cds1 *(S. cerevisiae* accession: P38221) provides CDP-diacylglycerol supply, which may be a precursor for phosphoinositide biosynthesis (in the plasma membrane), and as a negative regulator of phosphatidylinositol 4-kinase activity acting on cell proliferation via a lipid-dependent signal cascade (Shen et al. 1996; Shen and Dowhan 1997). Most *Mucorales* have 3 copies of this gene. This gene is also duplicated in *Mortierellomycotina*.

#### Phospholipid degradation

Phospholipid degradation recruits a variety of different enzymes. Lysophospholipase 1 PLB1 (*S. cerevisiae* accession: P39105), its paralog lysophospholipase 3 PLB3 (*S. cerevisiae* accession: Q08108) and lysophospholipase 2 PLB2 (*S. cerevisiae* accession: Q03674) are proteins that have grouped on the phylogenetic tree with a sequence of phospholipase B—meiotic phospholipase SPO1 (*S. cerevisiae* accession: P53541) (Tevzadze et al. 2000). Phospholipases B are required for efficient acyl chain remodeling of phosphatidylethanolamine-derived phosphatidylcholine (but not phosphatidylinositol) (Merkel et al. 2005a, b). Gene duplication is present in *Glomeromycotina* subphylum with a gene expansion in *Diversisporales.* There are also two copies present in the *Mucorales* order, except for the *Cunninghamellaceae* group and in *Endogonales* order. In Dikarya there are extensive gene expansions with 5 copies present in *Y. lipolytica* and 6 copies in *Aspergillus niger*. Polyphosphatidylinositol phosphatase INP52 (*S. cerevisiae* accession: P50942) dephosphorylates a variety of phosphatidylinositol phosphates to PI (Strahl and Thorner 2007). Gene loss has been observed in the *Endogonales*. Lysophospholipase NTE1 (*S. cerevisiae* accession: Q04958) is an intracellular phospholipase B catalyzing the double deacylation of phosphatidylcholine (PC) to glycerophosphocholine (GroPCho) (Murray and McMaster 2005). The protein also affects transcriptional repressor Opi1 localization (*S. cerevisiae* accession: P21957), regulating the genes involved in phospholipid biosynthesis (Fernández-Murray et al. 2009). Interestingly, the opposite action of Opi1 has been observed after phosphorylation by kinase A. The protein binds to PA and strongly correlates with overproduction of inositol (Sreenivas and Carman 2003). It seems to be *Saccharomycotina* specific and is not present in EDF.Mitochondrial N-acyl-phosphatidylethanolamine-hydrolyzing phospholipase D FMP30 (*S. cerevisiae* accession: Q02883) hydrolyzes N-acyl-phosphatidylethanolamines (NAPEs) to N-acylethanolamines (NAEs) and also is involved in maintaining proper cardiolipin level (Merkel et al. 2005a, b). The FMP30 gene has been lost in *Blastocladiomycota* and *Glomeromycota.* Gene duplication is present in *Mucorales*.

Glycerol kinase GUT1 (*S. cerevisiae* accession: P32190) is involved in glycerol degradation via glycerol kinase pathway, by converting glycerol to glycerol-3-phosphate. Its expression is also mediated by Opi1p (Grauslund et al. 1999). The GUT1 gene triplication is present in the *Mucoromycotina* subphylum.

Inositol phosphosphingolipids phospholipase C ISC1 (*S. cerevisiae* accession: P40015) hydrolyzes phosphosphingolipids (IPS), inositol phosphorylceramide (IPC), mannosylinositol phosphorylceramide (MIPC), and mannosyldiinositol phosphorylceramide (M(IP)2C) as well as regulates sphingolipid metabolism in mitochondria (Kitagaki et al. 2007; Sawai et al. 2000). Gene loss was observed in the *Blastocladiomycota* representatives while gene duplication was noted in the *Mucorinae* and *Phycomycetaceae* groups within the *Mucoromycotina* subphylum.

#### TAG and lipid bodies

**Fig. 6:**
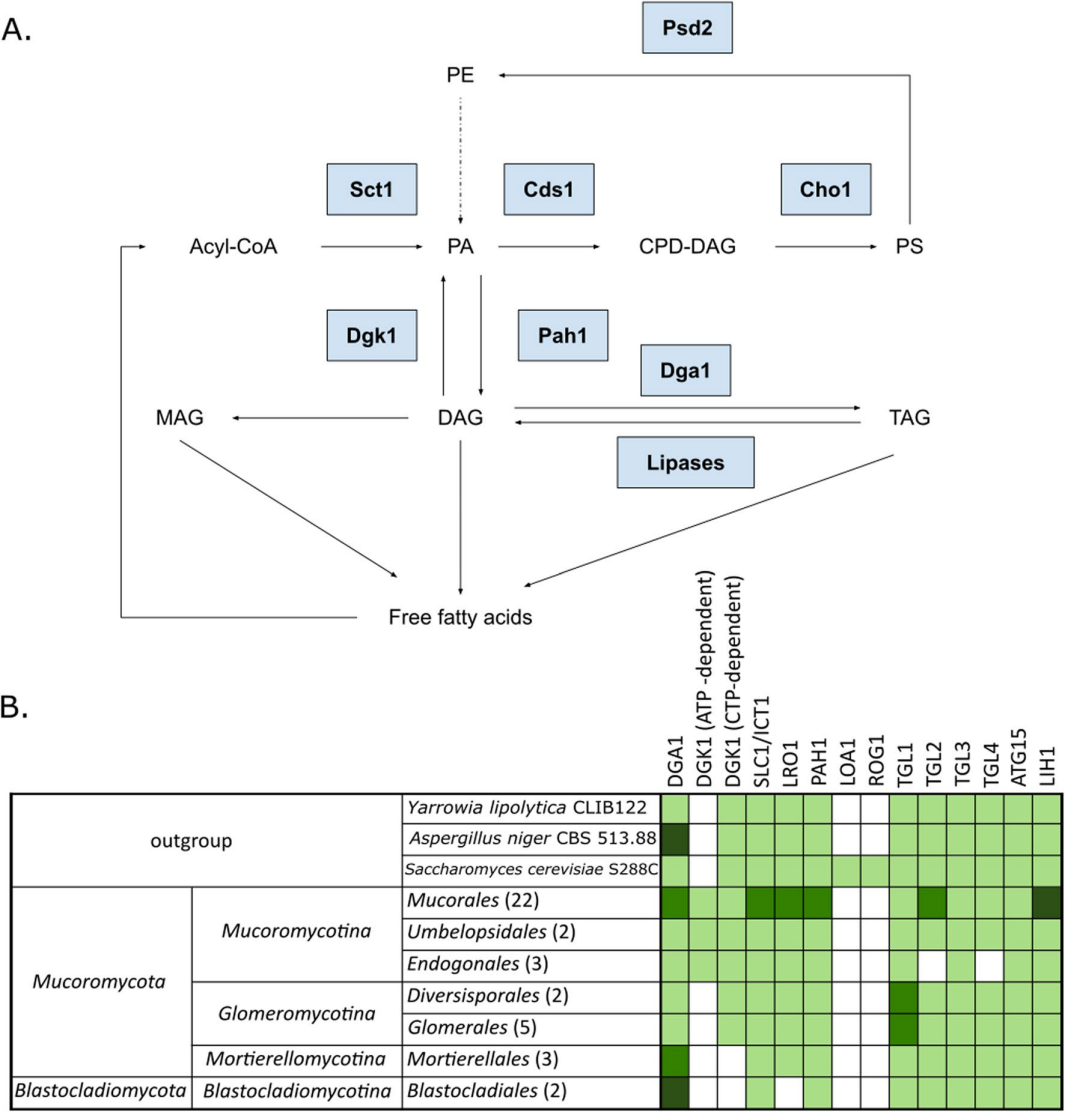
Metabolic map of TAG and lipid synthesis in EDF (**A**) and a table showing the distribution of genes encoding proteins involved in the TAG metabolism (**B**)—lighter green indicates presence of tested genes, middle green shows a duplication, while darker green indicates additional duplications. (*MAG* monoacylglycerol, *DAG* diacylglycerol, *TAG* triacylglycerol, *PE* phosphatidylethanoamine, *PA* phosphatidic acid, *PS* phosphatidylserine)

DAG derived from PA in phospholipid biosynthetic processes is also used for triacylglycerol (TAG) biosynthesis (Han and Carman 2004). Diacylglycerol O-acyltransferase 1 DGA1 (*S. cerevisiae* accession: Q08650) catalyzes the only committed step in the triacylglycerol (TAG) biosynthetic process (Fig. 6). It uses diacylglycerol and fatty acids as substrates for the TAG biosynthesis (Sorger and Daum 2002). Gene duplication occurred in the *Mucorales* order. Gene expansions are prevalent in the *Mortierellomycotina* and *Glomeromycotina* subphyla, as well as among the *Blastocladiomycota* (*Allomyces macrogynus* ATCC 38327) and Dikarya (*A. niger* CBS 513.88) representatives.

A homolog group of uncharacterized proteins with a domain architecture typical of bifunctional wax ester synthase/acyl-CoA: diacylglycerol acyltransferase consisting of WES_acyltransf (PF03007) followed by WS_DGAT_C (PF06974) was identified in the *Mucoromycota* phylum. These proteins are most similar to those found in uncharacterized bacteria (e.g. RME57737, AMS32783) and *Basidiomycota* proteins such as UMAG_01959 (XP_011387877) and presumably contribute to the formation of storage lipids like in *Acinetobacter calcoaceticus (*Kalscheuer and Steinbüchel 2003*)*.

Another central enzyme of the TAG metabolism is diacylglycerol kinase 1 DGK1 (*S. cerevisiae* accession: Q12382) that catalyzes the phosphorylation of diacylglycerol (DAG) to phosphatidate (PA). The enzyme is responsible for controlling the levels of PA and DAG synthesis for TAG biosynthesis and membrane phospholipids and is required for conversion of triacylglycerol-derived DAG to PA for phospholipid synthesis in the absence of de novo fatty acid synthesis (Han et al. 2008; Qiu et al. 2016). There are two types of fungal DGK enzymes: ATP-dependent and CTP-dependent. ATP-dependent diacylglycerol kinase (*Mucor circinelloides f. circinelloides* accession: S2JN77 and S2JXD0) is an ancestral protein consisting of two domains: DAGK_acc (PF00609) and DAGK_cat (PF00781). This DGK type is present in basal lineages of Holomycota (including *Fonticula alba* H696_03008 and *Capsaspora owczarzaki* CAOG_007051 with 3 paralogs), *Mucoromycotina* (in 2 copies), *Chytridiomycota* and *Kickxiellomycotina*, but not in Dikarya. CTP-dependent diacylglycerol kinase (*S. cerevisiae* accession: Q12382) consisting of DGK-like domain (IPR037997) is present in the SAR supergroup and many fungal lineages, including *Glomeromycotina*, *Basidiobolus* spp., *Mucoromycotina* and Dikarya, but absent in *Chytridiomycota*. Interestingly, after thorough tblastn searching Mycocosm database, none of the DGK types have been detected neither in *Mortierellomycotina* nor *Blastocladiomycota*, despite reports claiming the existence of DGK activity in the formers (Chang et al. 2022).

1-acyl-sn-glycerol-3-phosphate acyltransferase SLC1 (*S. cerevisiae* accession: P33333) catalyzes the sn-2-specific, acyl-CoA-dependent acylation of lysophosphatidic acid (LPA) to phosphatidic acid (PA) in lipid compounds (Athenstaedt and Daum 1997). On the phylogenetic tree it has grouped together with 1-acylglycerol-3-phosphate O-acyltransferase ICT1 (*S. cerevisiae* accession: Q12385), which is lysophosphatidic acid acyltransferase involved in remodeling of membranes, which leads to elevated organic solvent tolerance (Ghosh et al. 2008). Gene duplication is present in the *Mucorinae*.

Phospholipid: diacylglycerol acyltransferase LRO1 (*S. cerevisiae* accession: P40345) catalyzes the TAG formation by an acyl-CoA independent pathway, transfering acyl groups from phospholipids to DAG and forming an sn-1-lysophospholipid. The enzyme can also utilize ceramides instead of DAG, acylating those ceramides and creating 1-*O*-acylceramides (Dahlqvist et al. 2000); (Feng et al. 2019). *Blastocladiomycota* representatives lack the LRO1 gene in their genome while gene duplication was observed in the *Mucorales* order, in the *Mucorinae* group, and *Absidia* genus.

Phosphatidic acid phosphohydrolase 1 PAH1 (*S. cerevisiae* accession: P32567) is a Mg^2+^-dependent phosphatidate (PA) phosphatase responsible for the dephosphorylation of PA to DAG, essential for de novo lipid synthesis and formation of lipid droplets (Han et al. 2007). Gene duplication was identified in the *Mucorales* order, with additional copy present in the *Cunninghamellaceae* family.

Lipases hydrolyze the ester bond of tri-, di- and monoglycerides of long-chain fatty acids into fatty acids and glycerol. Analysis of triacylglycerol lipases TGL1-4 localized in lipid droplets (TGL1, TGL3 and TLG4) and in mitochondria (TGL2) have shown that TGL2 and TGL4 coding genes have been lost in the *Endogonales* order while gene duplication occurred in the TGL1 coding gene in the *Glomeromycotina* subphylum and in TGL2 gene in *Mucorales* order. Two putative lipases: ATG15 (*S. cerevisiae* accession: P25641) and LIH1 (*S. cerevisiae* accession: P47145) have grouped together on the phylogenetic tree (see Additional file 2: Dataset DS1 for all phylogenetic trees). ATG15 is required for the maintenance of lipid droplets and is also involved in the lysis of autophagic bodies (Maeda et al. 2015). Function of the LIH1 lipase is still unclear (Meunchan et al. 2015) and its multiple duplications have been identified in the *Mucorales* order*.*

### Sterol biosynthesis

**Fig. 7:**
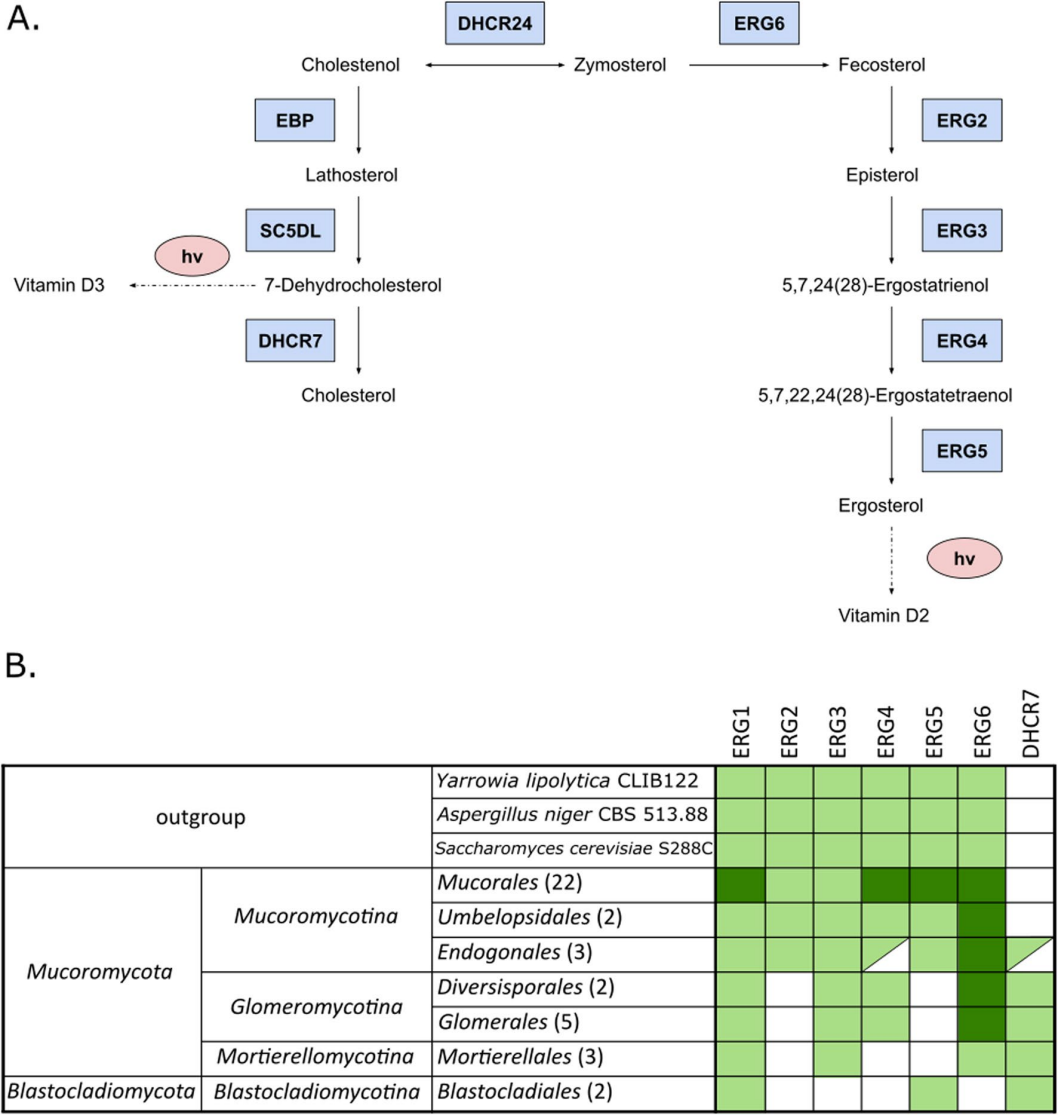
Schematic metabolic map of sterols in Fungi (**A**) and distribution of ERG genes in selected fungal lineages (**B**)—confirmed by BLAST against their genomic sequences in Mycocosm and NCBI. Lighter green indicates presence of tested genes, while darker green indicates additional duplications

There are five dominant end products of sterol biosynthesis, which are cholesterol, ergosterol, 24-methyl-cholesterol, 24-ethyl-cholesterol, and brassicasterol. Those compounds are considered the major sterols of fungi (Weete et al. 2010). One of the most studied steroid biosynthetic processes in fungi (KEGG pathway: map00100) is ergosterol biosynthesis. Ergosterol is an essential compound responsible for the fluidity and rigidity of fungal cell membranes and adaptation to the changing environment (Jordá and Puig 2020). Biosynthesis of ergosterol is a highly energy-consuming multistep process (Fig. 7).

This process starts with (R)-mevalonate biosynthesis from Acetyl-CoA. 3-hydroxy-3-methylglutaryl-coenzyme A reductase 1 HMG1 (*S. cerevisiae* accession: P12683) and its ohnolog-isoenzyme HMG2 (*S. cerevisiae* accession: P12684), catalyzing the conversion of HMG-CoA to mevalonate, which is regarded as a rate-limiting enzyme in the sterol biosynthesis (Basson et al. 1986). *Mucoromycota* genes are named HMGA (*P. blakesleeanus* accession: Q12649) and HMGR (*P. blakesleeanus* accession: A0A167JRE4), both bearing sterol-sensing domain and HMG-CoA_red domain. The Dikaryan HMG1 and HMG2 genes also code for proteins bearing the N-terminal domain with HPIH motif (PF00989), but the domain’s function is unknown.

The synthesis of ergosterol from zymosterol requires the consecutive activity of five enzymes labeled ERG2-6 and there is no compensatory pathway to synthesize ergosterol. Importantly, ergosterol can be converted spontaneously into vitamin D2 in the presence of light. There is however, a distinct pathway leading from 7-dehydrocholesterol (provitamin D3) to cholesterol present in animals and other opisthokonts (Fig. 7). Noteworthy, the provitamin D3 can be either converted spontaneously into vitamin D3 in the presence of light or reduced to cholesterol by 7-dehydrocholesterol-delta 7-reductase.

We have observed losses of ERG genes in lineages within *Mucoromycota* phylum. *Glomeromycotina* subphylum representatives have lost ERG2 (*S. cerevisiae* accession: P32352) and ERG5 (*S. cerevisiae* accession: P54781) genes while *Mortierellomycotina* subphylum representatives have additionally lost ERG4 (*S. cerevisiae* accession: P25340). *Endogone* sp. and *Jimgerdemannia flammicorona* are the only two within studied *Mucoromycotina* genomes in which we observed the loss of just the ERG4 gene. *Blastocladiomycota* phylum representatives lack ERG2, ERG3 (*S. cerevisiae* accession: P32353), ERG4, and ERG6 (*S. cerevisiae* accession: P25087) genes. This finding is consistent with the documented absence of ergosterol in *Glomeromycotina* and *Mortierellomycotina* (Weete et al. 2010).

Furthermore, in taxa: *Glomeromycotina*, *Mortierellomycotina*, *Blastocladiomycota,* and *Endogonales* we found 7-dehydrocholesterol-delta 7-reductase (*L. elongata* accession: OAQ35151), in humans coded by the DHCR7 gene. This protein is involved in the last step of cholesterol biosynthesis, which is the conversion of the 7-dehydrocholesterol (7-DHC) to cholesterol. This protein has been previously identified in *M. alpina* (Moebius et al. 1998; Wang et al. 2011; Zhang et al. 2007) and it was proved to be involved in the cholesta-5,24-dienol (desmosterol) production, which is the main sterol in *M. alpina* species.

Several fungal lineages showed duplications in genes coding ergosterol biosynthetic proteins (Table 1).

**Table 1:** Gene duplications of proteins involved in the ergosterol biosynthetic pathway observed within Mucoromycota phylum

Fungal clade	Duplicated genes	S. cerevisiae accession
*Glomeromycotina*	ERG6, ERG9	P25087, P29704
*Mucoromycotina*	ERG6	P25087
*Mucorales*	ARE1, ARE2, HMG1, HMG2, ERG1, ERG4, ERG5, ERG11, ERG20, ERG24, ERG25	P25628, P53629, P12683, P12684, P32476, P25340, P54781, P10614, P08524, P32462, P53045
*Umbelopsidales*	ERG25	P53045

Several fungal lineages showed duplications in genes coding ergosterol biosynthetic proteins, the most prominent group being *Mucorales*:

### Peroxisome and lipid degradation

The main function of fungal peroxisomes is beta-oxidation (KEGG pathway: map04146) of fatty acids (Maruyama and Kitamoto 2013). In yeast (*S. cerevisiae*), very long-chain fatty acids (VLCFAs) are activated in the cytosol by acyl-CoA synthases (Faa1 and Faa4) (Black and DiRusso 2007; Færgeman et al. 2001) and then transported to the peroxisomes by Pxa1- transporters Pxa2 and Fat1 (Hettema et al. 1996). Faa2 is responsible for the activation of medium-long-chain fatty acids (MLCFAs) directly in the peroxisomes (Deb and Nagotu 2017) while cell membrane-located Faa3 protein activates long-chain fatty acids (LCFAs; C16-C18) (Knoll et al. 1994). The proteins Faa1 (*S. cerevisiae* AC: P30624), Faa2 (*S. cerevisiae* accession: P39518) Faa3 (*S. cerevisiae* accession: P39002) and Faa4 (*S. cerevisiae* accession: P47912) are clustered together on the phylogenetic tree, which proves their shared evolutionary ancestry, however, the Faa2 protein is a branch clearly distant from Faa1, Faa3 and Faa4 proteins. Extensive gene expansions were observed in representatives of *Blastocladiomycota* (*Allomyces* sp. and *Catenaria* sp.) and duplications in the order *Mucorales* within the *Mucoromycota*. Fat1 protein (*S. cerevisiae* accession: P38225) is not present in members of *Blastocladiomycota*. Duplication of the FAT1 gene has been reported in *Mucorales* (with additional gene expansions) and *Umbelopsidales* within the *Mucoromycotina* subphylum and in representatives of *Mortierellomycotina*.

Beta-oxidation consists of four stages: dehydrogenation, hydration, oxidation, and thiolysis carried out by the enzymes Pox1, Fox2 and Pot1. The first step, oxidation of fatty acids, is conducted by the acyl coenzyme A Pox1 (*S. cerevisiae* accession: P13711) (Dmochowska et al. 1990). *Mucorales* have a duplication of the POX1 gene. Additionally, extensive gene expansion was observed in *Y. lipolytica*. The trifunctional protein Fox2 (*S. cerevisiae* accession: Q02207) with the properties of hydratase, dehydrogenase, and epimerase is responsible for the next step, which is the transformation of trans-2-enoyl-CoA, by D-3-hydroxyacyl-CoA into 3-ketoacyl-CoA. Duplication of the Fox2 gene occurred in all members of the *Mucoromycotina*. The last enzyme is Pot1 3-ketoacyl-CoA thiolase (*S. cerevisiae* accession: P27796), which cleaves the 3-keto-acyl-CoA molecule into acetyl-CoA and acyl-CoA (Mathieu et al. 1997). Duplication of the POT1 gene is present among members of the *Mucorales*. The 3–2-trans-enoyl-CoA isomerase Eci1 (*S. cerevisiae* accession: Q05871) is also involved in the isomerase-dependent beta-oxidation pathway for fatty acids with double bonds at unusual locations in the chain (Gurvitz et al. 1998). This enzyme is responsible for the conversion of cis-3-hexenoyl-CoA to trans-3-hexenoyl-CoA, which is a key step in beta-oxidation of unsaturated fatty acids (Gurvitz et al. 1998). This protein has a paralog in the form of delta (3,5)-delta (2,4)-dienoyl-CoA Dci1 isomerase (*S. cerevisiae* accession: Q08558), which is necessary for the proper peroxisomal localization of Eci1 (Gurvitz et al. 1999). Duplication of the gene encoding this isomerase is present among *Mucorales*.

#### Peroxisomal coenzyme A metabolism

Nudix hydrolases are a superfamily of enzymes capable of cleaving nucleoside diphosphates linked to x. They are associated with a number of different processes; mainly maintaining homeostasis, regulating the level of substrates in the cell (some of them are involved in peroxisomal beta-oxidation), and mRNA processing. Some of these enzymes are involved in peroxisomal CoA metabolism (Carreras-Puigvert et al. 2017; Hunt et al. 2014). These proteins are highly evolutionarily conserved among all organisms, but their function is still widely unknown (Carreras-Puigvert et al. 2017). In this paper we focus on the analysis of peroxisomal localized nudix superfamily proteins potentially involved in beta oxidation, i.e. Nudt7, Nudt12 and Nudt 19 (Carreras-Puigvert et al. 2017) Obtained results show the distribution of genes encoding nudix hydrolases among different fungal lineages and the evolutionary proximity of those gene sequences within the nudix superfamily.

**Fig. 8:**
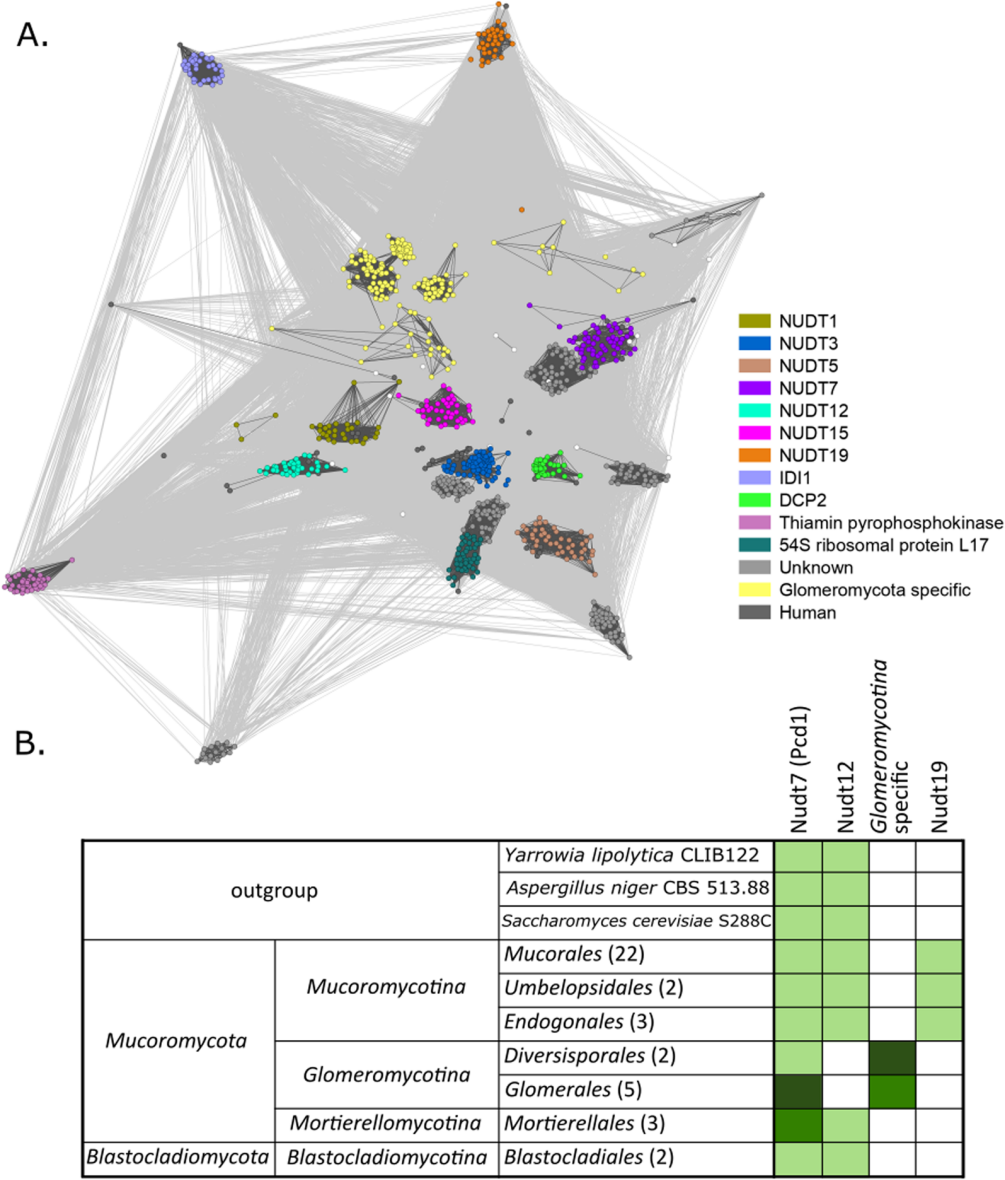
Visualization of the similarity of the amino acid sequences belonging to the NUDIX superfamily using the CLANS program (**A**) and distribution of NUDIX genes in selected fungal lineages (**B**). Lighter green indicates presence of tested genes, while darker green indicates additional duplications

Nudt7 (*H. sapiens* accession: P0C024), in yeast described as Pcd1 (*S. cerevisiae* accession: Q12524), is a coenzyme A diphosphatase that cuts free coenzyme A into 3', 5'-ADP and 4'-phosphopantetheine. This enzyme prefers oxidized CoA disulfides (CoAS-SCoA) as a substrate, which are potentially toxic and thus increase the cell's ability to beta-oxidize efficiently (Cartwright et al. 2000). Analysis of the Nudt7 protein sequence tree revealed duplication in the genomes of *Glomeromycotina* and *Mortierellomycotina*. Nudt12 (*H. sapiens* accession: Q9BQG2) hydrolyzes NADH and NADPH to reduced nicotinamide mononucleotide (NMNH) and AMP, usually acting on NAD-capped transcripts, but is also involved in peroxisome oxidative metabolism (Grudzien-Nogalska et al. 2019; Hunt et al., 2014b). The NUDT12 gene was not detected in the genomes of *Glomeromycotina* representatives, yet members of this group possess extensive duplications of genes encoding nudix hydrolases with unknown substrate specificity (Fig. 8, marked in yellow). They are especially numerous in *Gigaspora rosea* (n = 151) and *Diversispora epigaea* (n = 49). The Nudt19 enzyme (*H. sapiens* accession: A8MXV4) is coenzyme A diphosphatase, responsible for the hydrolysis of CoA esters. This hydrolase is specific for members of *Mucoromycotina* and is not present in other groups of fungi. During the clustering of protein sequences from the nudix family, many new families of nudix proteins with unknown substrate specificities and no homologs in humans were observed (Fig. 8, highlighted in light gray).

### Carotenoids

Among *Mucoromycota* carotenoid biosynthesis (KEGG pathway: map00906) is a unique feature of the *Mucoromycotina* representatives, absent in *Glomeromycotina* and *Mortierellomycotina* lineages. There are two enzymes involved in this pathway: bifunctional lycopene cyclase/phytoene synthase CarRP/carRA (*M. circinelloides* accession: EPB83040) and phytoene dehydrogenase CarB (*M. lusitanicus* accession: OAD07725). Only *Endogonales* order shows differences in distribution of genes coding these proteins; *Endogone* sp. have lost both CARRP and CARB genes and *Bifiguratus* sp. have lost the CARRP gene but *Jimgerdemannia flammicorona* has both genes.

## DISCUSSION

The basic lipid metabolism genes showed no significant diversity in distribution, however specialized lipid metabolic pathways differed in this regard among different fungal lineages. In total 165 out of 202 genes were present in all tested fungal lineages, while remaining 37 genes were found to be absent or lost in some of fungal lineages. Duplications were observed for 69 genes.

Early diverging fungi differ significantly in the ability to synthesize different types of lipids. Genomes of *Mucoromycotina* representatives are characterized by the whole genome duplication event (Stajich 2016), which explains many gene duplications observed in this subphylum. Especially in the *Mucorales* order, many copies of genes encoding lipid metabolism associated proteins were found. On the contrary, *Endogonales* representatives were found to have compact genomes, with multiple gene losses and very few gene duplications. Members of *Mortierellomycotina* possess numerous gene duplications in genes coding proteins involved in biosynthesis, elongation and desaturation of fatty acids. The same observations were made for *Glomeromycotina* representatives, except for the core fatty acid metabolism. In accordance with the findings made by Tang et al. (2016), *Glomeromycotina* representatives lack cytoplasmic fatty acid synthase (FAS-I) complex. This phenomenon is explained by the “Bread and Butter hypothesis” proposed by Rich et al (2017). The hypothesis suggests that arbuscular mycorrhizal fungi (*Glomeromycotina*) obtain both carbohydrates and lipids from their plant hosts. It seems that interactions with plants largely impact the lipidome of plant-associated fungi.

### Mucoromycota

The lipidome of *Mucoromycota* fungi seems to be characterized by the presence of delta-9 desaturase (*M. alpina* Uniprot accession: CAL69820), which allows those fungi to produce 18C polyunsaturated fatty acids, particularly gamma linolenic 18:3 fatty acid. Almost all *Mucoromycota* representatives (except for *Umbelopsidales* and *Endogonales*) possess duplications and expansions in the gene coding delta-9 desaturase.

A peculiar finding across the *Mucoromycota* phylum is the presence of proteins similar to diacylglycerol O-acyltransferases. These protein sequences show similarities to uncharacterized bacterial and *Basidiomycota* proteins. We speculate that these proteins could be involved in lipid storage, as in *Acinetobacter calcoaceticus* (Kalscheuer and Steinbüchel 2003).

Ancestral, animal-like heme peroxidases, have been observed in the proteomes of *Mucoromycota* representatives. Those enzymes seem to be evolutionarily related to the oxylipin desaturases present in Dikarya. They belong to a common protein family and share the same domain architecture as oxylipin desaturases, but differ in function significantly, in the sense that they don’t participate in lipid metabolism. Another animal-like feature observed in *Blastocladiomycota* and *Mucoromycota* is the presence of a pathway for converting 7-dehydrocholesterol (provitamin D3) to cholesterol. In most of the studied EDF species the presence of sphingomyelin-like synthase (*R. microsporus* Uniprot Accession: A0A1X0RK20) was found, along with sphingomyelin-like phosphodiesterase (*M. circinelloides* Uniprot Accession: S2JQ58), which is a homolog of the human Smpd1 protein that converts sphingomyelin to ceramides. The presence of sphingomyelin itself was already described by Bernat et al. (2018). This is a surprising finding since sphingomyelin is a lipid typical of animal cell membranes, previously thought to be absent from fungal cells (Huitema et al. 2004). The presence of those features among both EDF and animals probably indicates their ancestral origin and behavior in most representatives of both kingdoms.

### Mucoromycotina

The *Mucoromycotina* subphylum is composed of three orders that differ greatly in their ecology. However, some features of their lipidome are common. They possess the ancestral ATP-dependent diacylglycerol kinase (*Mucor circinelloides* f. *circinelloides* Uniprot Accession: S2JN77 and S2JXD0), absent in Dikarya, which possess only the CTP-dependent diacylglycerol kinase (*S. cerevisiae* Uniprot accession: Q12382). A characteristic feature of the representatives of *Mucoromycotina* is the combination of genes necessary for the production of beta carotene in one enzyme combining the functions of lycopene cyclase and phytoene synthase (Arrach et al. 2001). This type of protein was not detected in any other group of fungi. These proteins are extremely important as carotenoids play an important role in sexual reproduction in *Mucoromycota (*Lee and Heitman 2014; Schimek et al. 2003*)* and therefore are evolutionarily conserved. A specific coenzyme A diphosphatase belonging to the nudix family, a homolog of the human protein Nudt19 (*H. sapiens* Uniprot accession: A8MXV4), was detected in all members of this group. It is an enzyme specific to *Mucoromycotina* representatives, absent in all other groups of fungi.

The vast majority of fungi belonging to the *Mucorales* order are saprotrophs and pathogens of animals and plants (Spatafora et al. 2017). This group of fungi is characterized by genome-wide duplications, which explains numerous duplications and gene expansions that were observed in their genome (Stajich 2016). Their lifestyle is associated with the breakdown of dead matter, which requires capability to degrade various lipids, thus also explaining abundant duplications and expansion in lipids degradation pathways, as well as inositol signaling pathway.

*Endogonales* representatives are associated with plants, most of them with bryophytes. Their genomes are compact with many gene losses and a small number of gene duplications (Chang et al. 2019). The same observations apply to genes related to lipid metabolism.

Fungi belonging to the order *Umbelopsidales* are soil bound, most likely as saprophytic or rhizosphere-associated organisms, but some species are root endophytes (Hoff et al. 2004; Terhonen et al. 2014). *Umbelopsis isabellina* is a species that has been extensively studied for its capability to biosynthesize mono- and polyunsaturated fatty acids (Papanikolaou and Aggelis 2019). Few gene duplications were detected in genomes of *Umbelopsidales*, mainly in genes encoding fatty acid elongases and Maw3 desaturase, which may explain their ability to accumulate industrially desirable PUFAs.

### Glomeromycotina

*Glomeromycotina* subphylum consists of obligate symbionts of terrestrial plants (Bruns et al. 2018; Spatafora et al. 2017). Many adaptations to the strictly symbiotic lifestyle have been observed in their lipidomes. Among the representatives of *Glomeromycotina*, the loss of genes encoding proteins related to the core lipid metabolism, cytoplasmic fatty acid synthase (both Fas1 and Fas2 subunits) was confirmed. On its basis the "bread and butter" hypothesis was created (Rich et al. 2017), claiming that arbuscular fungi, apart from carbohydrates, receive simple fatty acids from the host plant and therefore the fatty acid biosynthetic pathway is redundant for the symbiote. In addition to the loss of fatty acid synthase, they have lost peroxisomal acyl-coenzyme A Tes1 thioesterase (*S. cerevisiae* Uniprot accession: P41903) that hydrolyzes acyl-CoAs to free fatty acids and CoASH (Jones et al. 1999), which also appears to be related to their symbiotic lifestyle.

Despite their inability to synthesize basic fatty acids, members of *Glomeromycotin*a possess the capability to produce a wide variety of complex, long-chain fatty acids, including many PUFAs. Numerous duplications were observed in the genes encoding proteins responsible for the elongation and desaturation of fatty acids. One such duplicate desaturase is the acyl CoA Ole1 desaturase. Probably one of the duplicate proteins has a different activity related to the production of palmitvacenoic acid (Δ11-cis-palmitvacenoic acid), which is produced only by representatives of *Glomeromycotina* (Brands et al. 2020). *Glomeromycotina* also have the ability to synthesize arachidonic acid with a length of 20 carbon atoms, due to the presence of delta-5 desaturase.

A unique type of nudix hydrolases has also been observed in the lipidome of *Glomeromycotina*. They lack the gene encoding Nudt12 protein, however extensive duplications of the genes encoding nudix hydrolases with unknown substrate specificity were found. *Diversisporales* representatives that were found to have multiple duplications of these sequences are *Gigaspora rosea* (n = 151) and *Diversispora epigaea* (n = 49). It is possible that these hydrolases are associated with the removal of mutations in multinucleated cells or with oxidative stress. This phenomenon can also be explained by the fact that so far no sexual reproduction has been observed in representatives of this group of fungi (Jany and Pawlowska 2010). Consequently, repairing replication errors is extremely important for maintaining homeostasis inside the cell.

An ancestral feature of *Glomeromycotina* subphylum is the presence of 24-ethyl-cholesterol instead of ergosterol in their cell membranes. Weete et al. (2010) have observed the lack of a functional pathway for ergosterol synthesis, which can be further explained by the lack of genes encoding the Erg2 and Erg5 enzymes (Amses et al. 2022). However, presence of a unique for fungi, probably ancestral, pathway converting 7-dehydrocholesterol (provitamin D3) to cholesterol associated with the presence of the protein Dhcr7 was noted in *Glomeromycotina (*Amses et al. 2022*)*. In addition, the presence of acyl-AMP ligases (FAALs), was detected, originally discovered in *Mycobacterium* sp. Their function is to convert fatty acids to adenylates, which are substrates for acyl-CoA-synthesizing acyl-CoA ligases (acyl-CoA-synthesizing fatty acyl-CoA ligases; FACLs) (Arora et al. 2009).

### Mortierellomycotina

In terms of the fatty acid metabolism, *Mortierellomycotina* representatives show similar characteristics to *Glomeromycotina*. This is not a surprising finding as both fungal groups interact with plants, therefore similarities in terms of their ability to synthesize lipids are expected. Unlike *Glomeromycotina, Mortierellomycotina* possesses all the genes encoding proteins related to the synthesis of basic fatty acids. A surprising observation is the lack of diacylglycerol kinase (Dgk1). It has been reported that the Dgk protein is present in *Mortierellomycotina* (Chang et al. 2022), but without any reference to specific gene or sequence identifiers described. The results obtained in this study suggest that the gene encoding diacylglycerol kinase is not present in the genomes of this group. This is a surprising finding and, if true, means that there is an alternative way to regulate the levels of PA and DAG synthesis, which is necessary to maintain the synthesis homeostasis of phospholipid metabolism.

A distinctive feature of the fungal lipidome of the *Mortierellomycotina* is the ability to produce polyunsaturated fatty acids with a length of up to 20 carbon atoms in the chain, of which the most important is arachidonic acid. This is a well-documented capacity associated with the delta-5 desaturase present only in *Blastocladiomycota, Mortierellomycotina,* and *Glomeromycotina* (Chang et al. 2022). Duplication of the Maw3 gene, which is involved in the synthesis of other C20 acids, has also been observed (Chang et al. 2022).

*Mortierellomycotina* also lack ergosterol in their cell membranes. The different composition of sterols in the cell membranes of representatives of *Mortierella* spp. was documented as early as 1996 (Weete and Gandhi 1996) but the molecular basis of this phenomenon has not been established so far. In this study it was proved that representatives of *Moriterellomycotina* have lost the ERG2, ERG4 and ERG5 genes and therefore are unable to produce ergosterol. In addition, similarly to *Glomeromycotina*, an atypical animal pathway was also observed to convert 7-dehydrocholesterol (provitamin D3) to cholesterol, associated with the presence of the protein Dhcr7. This protein was previously identified and characterized by Zhang et al. (2007) and Wang et al. (2011) as the enzyme involved in desmosterol production. We suggest, however, that this protein is widely present among plant-associated *Mucoromycota* species, which tend to accumulate cholesterol derivatives instead of ergosterol, as their main cell membrane sterol.

## CONCLUSIONS

Based on the analysis of duplications, expansions, and losses of genes in early diverging fungi, we reconstructed the evolutionary history of proteins involved in lipid metabolism and linked it to the ecology of different fungal groups. Plant-associated *Glomeromycotina*, *Mortierellomycotina*, and *Endogonales* representatives have lost some of the enzymes involved in ergosterol synthesis, however they possess an ancestral pathway leading to the synthesis of cholesterol, which is absent in other fungal lineages. *Glomeromycotina* and *Mortierellomycotina* are also characterized by the similar set of desaturases and elongases, which are involved in the synthesis of polyunsaturated fatty acids. We also identified a complete loss of genes encoding for any type of diacylglycerol kinases in *Blastocladiomycota* and *Mortierellomycotina*. In the proteomes of *Glomeromycotina* we identified a wide repertoire of NUDIX family hydrolases of unknown substrate specificity. Different, ancestral type of NUDIX hydrolases, not found in any other fungal lineage, was also identified in the proteomes of *Mucoromycotina*. Moreover, evolutionary solutions present in animals, such as the presence of animal-type heme peroxidases or sphingomyelin related proteins, have been observed in the proteomes of representatives of *Mucoromycota*, which suggests an ancient origin of these traits.

## METHODS

### Curation of the set of lipid metabolism genes

To investigate the evolutionary histories of families of genes involved in lipid metabolism across *Mucoromycota* we curated a dataset of genes from *Saccharomyces cerevisiae* in the KEGG diagrams of lipid metabolism pathways. Additionally lipid metabolism genes were collected from literature assisted with database searches MetaCyc (Caspi et al. 2018), UniProt (Consortium and The UniProt Consortium 2019), BRENDA (Schomburg et al. 2017), KEGG (Kanehisa et al. 2017), SGD (Chan and Cherry 2012) and STRING (Szklarczyk et al. 2017) (Additional file 1: Table S1). MetaCyc and KEGG metabolic maps were used as a starting point. Genes from key lipid pathways in *S. cerevisiae* were used also in STRING in transitive graph navigation to ensure the whole interaction network is present. *Saccharomyces cerevisiae* core metabolism gene list was extended by genes known from *Mucor circinelloides* (Tang et al. 2015), *Yarrowia lipolytica* (Lazar et al. 2018), and *Mortierella alpina* (Chang et al. 2022). UniProt fungal protein annotations were queried online with the list of keywords used to find fungal genes involved in lipid metabolism including “lipid metabolism”, “fatty acid biosynthesis”, “lipid biosynthesis”, “lipid degradation”, “ceramide”, “phospholipid”, “glycolipid”, “ergosterol”, “sterole”, “wax”, “PUFA biosynthesis”, “beta-oxidation”,, “triacylglycerol”, “sphingolipid”, “carotenoid”, “FAS”.

Metabolic maps were also inspired by Cassilly and Reynolds (2018), Singh and Del Poeta (2016), Kikukawa et al. (2018), Lastovetsky et al. (2016).

### Genomic dataset and sequence analyses

Forty predicted non-Dikarya and three *Ascomycota* proteomes were downloaded from NCBI in March 2021 (Sayers et al. 2020) (Additional file 1: Table S2) and searched with blastp (e-value threshold—1e-25, coverage threshold 40% and identity threshold 30%) with the list of known fungal lipid metabolism genes. Homologs of each gene were aligned using local iterative mode in Mafft v. 3.7 (maxiterate = 100) (Katoh et al. 2019), subsequently alignments were trimmed with TrimAl (model = gappyout) (Capella-Gutiérrez et al. 2009) and subjected to phylogenetic tree analysis using a Maximum likelihood (ML) approach. IqTree v 1.6.9 with automated model selection was used to build gene trees (-m MFP -alrt 0 -t RANDOM -nt AUTO) (Minh et al. 2020).

Orthologous protein coding genes were identified using Orthofinder 2 (Emms and Kelly 2019) with Diamond (Buchfink et al. 2015). In total, 33,600 orthogroups were found including 667 in which all of the species were present. DendroBLAST was used to create unrooted trees for each orthogroup (Kelly and Maini 2013). The final species tree was inferred with STAG (Emms and Kelly 2018) and rooted with STRIDE (Emms and Kelly 2017). It was then compared to the most recent phylogenomics tree of fungi (Li et al. 2021).

Each gene tree was then reconciled with the species tree in Notung with default DL parameters (Stolzer et al. 2012) and visually in iToL (Letunic and Bork 2021).

Protein architecture was annotated with a pfam_scan.pl (default settings) (Mistry et al. 2007) search against Pfam v.34 library of HMMs (Mistry et al. 2021). Gene trees were visualized in iTol with protein annotations as datasets (Letunic and Bork 2021).

Protein annotations were checked against OrthoDb (Zdobnov et al. 2021) and human uniprot accessions when fungal homologs were consistently labeled or missing due to absence/loss in Dikarya. This was particularly often in the case of sphingolipids and oxidation related homologs.

### Supplementary Information

**Additional file 1:** Spreadsheet with gene names, protein accessions and list of analysed fungal assemblies.

**Additional file 2:** Phylogenetic trees of all proteins.

## Data Availability

All accessions and assemblies are listed in Additional file 1: Table S1 and all the phylogenetic trees are available as a text file Additional file 2: Dataset DS1.
